# Determinants of Initiation Codon Selection during Translation in Mammalian Cells

**DOI:** 10.1371/journal.pone.0015057

**Published:** 2010-11-24

**Authors:** Daiki Matsuda, Vincent P. Mauro

**Affiliations:** Department of Neurobiology, The Scripps Research Institute and The Skaggs Institute for Chemical Biology, La Jolla, California, United States of America; Victor Chang Cardiac Research Institute, Australia

## Abstract

Factors affecting translation of mRNA contribute to the complexity of eukaryotic proteomes. In some cases, translation of a particular mRNA can generate multiple proteins. However, the factors that determine whether ribosomes initiate translation from the first AUG codon in the transcript, from a downstream codon, or from multiple sites are not completely understood. Various mRNA properties, including AUG codon-accessibility and 5′ leader length have been proposed as potential determinants that affect where ribosomes initiate translation. To explore this issue, we performed studies using synthetic mRNAs with two in-frame AUG codons−both in excellent context. Open reading frames initiating at AUG1 and AUG2 encode large and small isoforms of a reporter protein, respectively. Translation of such an mRNA in COS-7 cells was shown to be 5′ cap-dependent and to occur efficiently from both AUG codons. AUG codon-accessibility was modified by using two different elements: an antisense locked nucleic acid oligonucleotide and an exon-junction complex. When either element was used to mask AUG1, the ratio of the proteins synthesized changed, favoring the smaller (AUG2-initiated) protein. In addition, we observed that increased leader length by itself changed the ratio of the proteins and favored initiation at AUG1. These observations demonstrate that initiation codon selection is affected by various factors, including AUG codon-accessibility and 5′ leader length, and is not necessarily determined by the order of AUG codons (5′→3′). The modulation of AUG codon accessibility may provide a powerful means of translation regulation in eukaryotic cells.

## Introduction

Translation initiation involves at least two primary processes, ribosomal recruitment and recognition of an initiation codon. For some mRNAs, the recruitment site lies in close proximity to the initiation codon and effectively links these two processes. Examples include the Shine-Dalgarno interaction in *E. coli* [1] and an internal ribosome entry site (IRES) in Cricket Paralysis virus RNA [2], [3]. However, in eukaryotes, ribosomal recruitment generally occurs some distance upstream of the initiation codon, at either the 5′ m7G cap-structure or an upstream IRES [4], [5], [6]. This spatial arrangement requires ribosomal subunits to move from the recruitment site to the initiation codon, which, depending on the mRNA, may be at the first AUG codon, a downstream AUG codon, or multiple AUG codons. In some cases, translation initiates at an alternative initiation codon, such as ACG, CUG, or GUG [7], [8]. For example, a recent study in yeast revealed that ≈20% of ribosome footprints in 5′ leaders were due to the translation of upstream ORFs that initiate via non-AUG codons [9]. Nucleotides flanking an initiation codon can also affect its efficiency, as can the length and structural stability of the 5′ leader [4], [10], [11], [12]. However, the effects of these various features are not easily predicted since we have an incomplete understanding of the molecular details of ribosomal movement during translation initiation.

Several recent studies have used bioinformatic and computational approaches to investigate features in mRNAs that affect translation initiation. For example, a computational analysis of the genomes of 340 species found that RNA structural stability is predicted to be reduced immediately downstream of initiation codons, in both prokaryotic and eukaryotic mRNAs [13]. The authors suggested that such reduced stability is a universal feature of mRNAs and is more likely to affect initiation codon recognition than ribosomal recruitment. In both *E. coli* and *S. cerevisiae*, a general trend of weaker folding stability was predicted to occur in the regions surrounding initiation codons [14]. In addition, a study in *E. coli* showed that synonymous mutations in the coding region of *green fluorescent protein* mRNA affected protein expression by up to 250-fold [15]. Remarkably, the major variable affecting expression in this study was predicted reduced stability in the region surrounding the initiation codon (−4 to +37; where the A of the AUG codons is +1). In an earlier study, we postulated that the relative accessibility of the initiation codon in eukaryotic mRNAs may affect its ability to base pair to the initiator Met-tRNA and initiate translation [4].

These findings prompt a further analysis of the effects of initiation codon accessibility on translation initiation in eukaryotes. In the present study, we directly test the hypothesis that the relative accessibility of an AUG codon affects its use as an initiation codon in mammalian cells. Our experiments were performed using synthetic mRNAs with the following features: 5′ leaders were designed to have a low propensity to form stable secondary structures; they contained two in-frame AUG codons in excellent context; and their translation was cap-dependent. To diminish nucleotide accessibilities at specific sites *in vivo*, we used two different masking elements: antisense locked nucleic acid (LNA) oligonucleotides and exon-junction complexes (EJCs). These two elements were chosen for this study because they can mask specific sites in mRNAs by different mechanisms. An LNA oligonucleotide can base pair stably to complementary nucleotides [16] and thereby mask them. By contrast, an EJC is a protein complex that is deposited upstream of exon-exon junctions [17], and can mask nucleotides in a sequence independent manner. The results indicate that decreasing the accessibility of the first AUG codon by using either masking element reduces translation initiation at this codon. The data provide experimental support for the hypothesis that AUG codon accessibility is an important variable in determining where translation initiates. In addition, we observed that other factors, including the length of the 5′ leader, its nucleotide composition, and the cell line could also alter the ratio of AUG codon usage. These findings indicate that the selection of an AUG codon depends on numerous variables, alteration of which can redirect translation initiation.

## Methods

### DNA constructs

**Figure 1:**
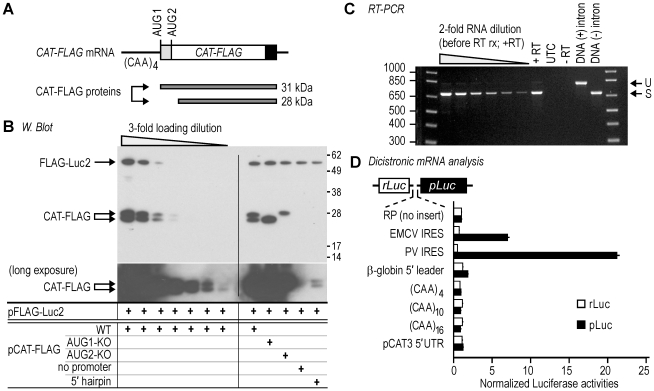
Translation of a synthetic *CAT-FLAG* mRNA initiates at two AUG codons in a cap-dependent manner. **A**. The *CAT-FLAG* mRNA used in this study is indicated schematically. It contains four CAA repeats in the 5′ leader and two in-frame AUG codons. FLAG epitopes are indicated by the black bar. This mRNA encodes two proteins with predicted molecular weights of 31 and 28 kDa. **B**. Western blot analysis. COS-7 cells were transiently cotransfected with plasmid constructs that express the *(CAA)_4_ CAT-FLAG* and the control *FLAG-Luc2* mRNAs. The wild-type CAT-FLAG construct (WT) contains both AUG codons; AUG1-KO lacks AUG1 (the U has been deleted); and AUG2-KO lacks AUG2 (mutated to AAG). The no promoter construct lacks SV40 promoter/enhancer sequences and the 5′ hairpin construct contains an inverted repeat sequence at the 5′ terminus of the mRNA. A longer film exposure of the blot is shown for the CAT-FLAG protein. **C**. RT-PCR analysis of *(CAA)_4_ CAT-FLAG* mRNA from COS-7 cells transfected with a plasmid expressing this mRNA (+RT). Control reactions used RNA from untransfected cells (UTC) or did not contain reverse transcriptase (-RT). Size controls for PCR products of unspliced (U) or correctly spliced (S) mRNAs were amplified from plasmids containing an intron (DNA (+) intron) or lacking an intron (DNA (-) intron), using the same PCR conditions in parallel. Two-fold dilutions of the *(CAA)_4_ CAT-FLAG* RNA sample were reverse-transcribed prior to PCR amplification. **D**. *Renilla/Photinus* dual luciferase dicistronic analysis. The 5′ leader sequences were tested in the intercistronic region of the dicistronic mRNA. Intercistronic sequences in the parent vector (RP) and the *β-globin* 5′ leader were used as negative controls for IRES and promoter activities; the EMCV and PV 5′ leaders were used as positive controls for IRES activity. *Renilla* luciferase (rLuc) activities are indicated by white bars; *Photinus* luciferase (pLuc) activities are indicated by black bars. Luciferase activities were normalized to 1.0 for activities obtained with the RP construct. Three independent experiments were performed for final quantification; error bars indicate standard deviations.

Synthetic mRNAs for these studies contain two in-frame AUG codons (AUG1 and AUG2) and encode a chloramphenicol acetyltransferase (CAT) protein having three copies of a FLAG epitope tag at its C-terminus (**Figure 1A**). The *CAT* gene for these constructs was derived from the pCAT3-Control plasmid (Promega) and the FLAG sequence was from p3XFLAG-CMV-7 plasmid (Qiagen). An ATG (AUG1) was introduced into the vector-derived 5′ leader, in-frame with the authentic CAT initiation codon to generate a CAT-FLAG protein with a 26 amino acid N-terminal extension (see Methods S1 for sequences). This sequence corresponds to nucleotides 275-482 in the pCAT3-Control vector, excluding the 133-nt chimeric intron. The two encoded proteins can be differentiated by Western blot analysis. The nucleotide contexts of the two AUG codons are AAG**AUG**GG for AUG1 and ACC**AUG**GA for AUG2. The resulting *CAT-FLAG* gene was cloned into pGL4.13 (Promega), replacing the *luc2* gene, and was transcribed via the SV40 promoter. 5′ leader sequences upstream of AUG1 include variable numbers of (CAA) repeats and *β-globin* 5′ leader sequences, which were expected to be relatively unstructured. These 5′ leader sequences were generated by PCR and subsequently cloned into the plasmid vector (see Methods S1 for details of 5′ leader sequences). For constructs with a 5′ hairpin structure, a 66-nt sequence containing an inverted repeat capable of forming a stable stem-loop structure (?G  =  -67.6 kcal/mol) was inserted immediately downstream of the SV40 promoter using the *Aat*II and *Eco*RI restriction sites. The intron from pCAT3-Control, which contains consensus splice donor, acceptor, and branch sites, was inserted 69 nucleotides downstream of AUG2 to enhance mRNA expression. Promoterless constructs were generated by deleting the SV40 promoter by blunt-end ligation after digestion with *Eco*RV and *Stu*I. PCR products containing the 5′ leader sequences of *CAT-FLAG* genes (upstream of AUG2) were cloned into plasmid pGL3-R2 (RP) [18], in the intercistronic region of a *Renilla* luciferase-*Photinus* luciferase dicistronic gene using *Eco*RI and *Nco*I restriction sites. To direct EJC deposition on AUG1 or AUG2 upon mRNA maturation, the chimeric intron used in the constructs described above was relocated 23- or 19-nucleotides downstream of AUG1 or AUG2, respectively. These constructs contain the full-length 5′ leader sequence from pCAT3-Control and are designed to be identical in primary sequence upon splicing.

A cotransfection control construct for these studies encodes a FLAG-tagged *Photinus* luciferase gene (*FLAG-Luc2*). This gene was generated using the *Luc2* gene (Promega), which was PCR-amplified from pGL4.13 using a forward primer containing an in-frame FLAG tag sequence. The PCR product was cloned into pCI-neo (Promega) using *Eco*RI and *Xba*I.

### In vitro transcription of capped mRNAs

For RNA transfections, *in vitro* transcripts were generated using intron-free *CAT-FLAG* reporter genes containing the T7 promoter sequence upstream of the 5′ leaders. These reporter genes were generated by using PCR and the products cloned into pGL4.13 with *Bgl*II and *Xba*I restriction sites. A 70-nt stretch of poly(A) was introduced 167 nucleotides downstream of the *CAT* termination codon. This site of poly(A) addition is the same as that for mRNAs expressed in cells from comparable pGL4.13-based plasmids [19]. Capped *in vitro* transcripts were generated by using mMessage mMachine (Ambion) from these plasmids linearized with *Bam*HI, which is located immediately downstream of the poly(A)_70_ sequence. mRNAs were quantified by UV absorption at 260 nm, and mRNA quality evaluated by 1% agarose gel electrophoresis.

### LNA antisense oligonucleotides

Among the elements used in this study to reduce AUG codon accessibility were LNA oligonucleotides, which contain modified ribose sugars in which the 2′-O and 4′-C atoms are linked via a methylene group that locks the ribose conformation and confers high binding affinity to a complementary sequence [16] as well as high nuclease-resistance [20]. Oligonucleotides used in this study were fully modified with LNA nucleotides. (LNA-AUG1 13-mer: 5′-GACCCATCTTCTG-3′ and 9-mer: 5′-GACCCATCT-3′) were designed to target AUG1 in reporter mRNAs. An isosequential LNA oligonucleotide LNA-C (5′-CGACTTCCTACTG-3′) was generated by scrambling the LNA-AUG1 sequence and was used both as a negative control for the antisense effect, and as a specific oligonucleotide to target the 5′ leader sequence upstream of AUG1 in appropriate mRNA constructs. LNA oligonucleotides were purchased from Eurogentec.

### Transfection

COS-7 cells were grown and passaged as described previously [21]. The cells were seeded in 6-well plates at 1.2×10^5^ cells/well and transfected the next day with plasmids using FuGENE 6 (Roche Diagnostic), or with plasmids and LNA oligonucleotides using Lipofectamine 2000 (Life Technologies), according to manufacturer's recommendations. Cells were cotransfected with 0.4 µg of reporter plasmid, which expresses the *CAT-FLAG* gene and with 0.05 µg of the *FLAG-Luc2* cotransfection control plasmid. For transfections that included LNA oligonucleotides, the amounts used are specified in the text and ranged up to 300 nM for the 13-mer oligonucleotides and 3 µM for the 9-mer oligonucleotide. Transfected cells were harvested 18–22 h after transfection with 150 µl 1x Passive Lysis Buffer (Promega) for protein analysis or 1 ml Trizol reagent (Life Technologies) for RNA extraction and primer extension reactions. One third of the lysate in Passive Lysis Buffer was treated with 200 µl Trizol reagent and chloroform to extract RNA for reverse transcription (RT)-PCR. Isopropanol-precipitated RNA was dissolved in double distilled water (6-10 µl) and stored at −80°C.

For RNA transfections, COS-7 cells were seeded at 2.5×10^5^ cells per well in 6-well plates and transfected the next day using Lipofectamine 2000 with 1 pmol of *in vitro* transcribed capped mRNA in the presence or absence of 10 pmol of LNA antisense or control oligonucleotides. At 1 h post-transfection, media were exchanged with FBS-containing DMEM and cells were further incubated and then harvested with 100 µl 1x Passive Lysis Buffer at the time specified in the text. One third of the lysate along with any cells left in the wells were treated with 200 µl Trizol (Life Technologies) to extract total RNA for primer extension.

### Primer extension analyses and semi-quantitative RT-PCR

One third of the Trizol-extracted RNAs were used for primer extension analysis (5 µl reaction volume) using AMV reverse transcriptase (Life Technologies). The extracted RNA and dNTPs (5 nmol each) were heated to 90°C for 3 min and immediately placed on ice. A 5′-end ^32^P-labeled primer (≈0.2 pmol; >40,000 cpm) was then added to the RNA along with 1x cDNA Synthesis Buffer (Life Technologies), 25 nmol DTT and 4U murine RNase inhibitor (NEB), and incubated at 37°C for 5 min. Subsequently, AMV reverse transcriptase (4U) was added, and the reaction was further incubated at 37°C for 30 minutes. The reaction was terminated by adding 5 µl of Gel Loading Buffer II (Ambion) and resolved in 6% denaturing PAGE. RNA samples for semi-quantitative PCR were treated with DNase by using the DNA-Free Turbo kit (Ambion); reverse transcription reactions were then performed using random hexamers and Superscript III (Life Technologies) by following manufacturer's directions. See Methods S1 for primer details.

### Analyses of Reporter Gene Expression

Cell lysates in 1x Passive Lysis Buffer were analyzed for *Photinus* and/or *Renilla* luciferase activities from transfected cells as described previously [18], [22]. Western immunoblotting analyses of CAT-FLAG expression were performed as previously described [21], using 10% or 4–12% gradient Bis-Tris polyacrylamide gels (Life Technologies) and monoclonal anti-FLAG M2 antibody (Sigma). Serial dilutions of cell lysates were electrophoresed in parallel to allow quantification of the relative abundance of each protein. Quantification was performed on scanned images using ImageQuant software (Molecular Dynamics). For statistical analysis, two-sample t-test or one-way ANOVA was used to determine p-values; p-values < 0.05 are considered statistically significant difference and indicated in the figures where applicable.

### Northern Blot Analyses

Northern blots were performed as described previously [18] using ≈30 µg of total RNA from COS-7 cells for poly(A) selection. Hybridizations were performed using 5′-end ^32^P-labeled DNA oligonucleotides in ULTRAhyb Ultrasensitive Hybridization Buffer (Ambion) at 37°C overnight. The *β-globin CAT-FLAG* mRNAs were detected by using various probes to hybridize either upstream or downstream of the LNA-AUG1 binding site in the mRNA (see Methods S1 for sequence of the probes). The control *FLAG-Luc2* mRNA was detected on the same membrane by subsequent hybridization of membranes with probes that are specific to this mRNA.

## Results

To investigate accessibility as a variable affecting initiation codon usage, we developed a synthetic mRNA that initiates translation by a cap-dependent mechanism. We then used two elements to mask different sites in this mRNA: antisense LNA oligonucleotides to mask nucleotides by stably base pairing to them and EJCs to mask nucleotides in a sequence independent manner. The following experiments describe functional characterization of the synthetic mRNA and the use of LNA oligonucleotides and EJCs as masking elements. In addition, numerous control experiments were performed to test alternative possible explanations for the data.

### Synthetic mRNA directs cap-dependent expression of two proteins

For this study, we developed a synthetic mRNA that contains the *CAT* cistron (**Figure 1A**). To facilitate immunoblot detection of the full-length protein, sequences encoding three copies of a FLAG peptide tag were appended at the C-terminus. The 5′ leader contains four tandem repeats of the trinucleotide CAA. This repeat was chosen because the resulting sequence appears to be unstructured and does not contain AUG or alternative initiation codons [23]. The *CAT-FLAG* mRNA was designed with two in-frame AUG codons, which we refer to as AUG1 and AUG2 (5′→3′), and which are separated by 78 nucleotides of vector-derived sequence. The two in-frame overlapping open reading frames (ORFs) differ by 26-amino acids at the amino termini and encode proteins with predicted molecular weights of 31 and 28 kDa. Nucleotides at critical positions relative to AUG1 and AUG2 are optimized for translation initiation. These nucleotides are an A at position -3 and a G at position +4. COS-7 cells transfected with a construct expressing this mRNA produced two proteins detectable by immunoblotting with an anti-FLAG antibody (**Figure 1B**; WT). The two proteins were present at similar levels and their sizes (≈28 and 25 kDa) appeared to be slightly smaller than those encoded by the mRNA. However, the origin of the proteins was confirmed by showing that the larger protein was not expressed when AUG1 was deleted (**Figure 1B**; AUG1-KO), and the shorter product was not expressed when AUG2 was deleted (**Figure 1B**; AUG2-KO).

Control experiments were performed to determine whether the smaller protein in cells transfected with the parent construct was translated from full-length mRNAs containing both AUG codons, or from shorter mRNA species lacking AUG1. To exclude whether a shorter mRNA is transcribed from a cryptic promoter, we deleted the SV40 promoter/enhancer in the (CAA)_4_ CAT-FLAG construct. The results show that expression of both CAT-FLAG proteins was decreased to an undetectable level (**Figure 1B**; no promoter, long exposure), ruling out cryptic promoter activity and suggesting that both proteins are expressed from SV40-driven (full-length) transcripts. Additional evidence supporting this conclusion is the finding that the introduction of a stable stem-loop structure at the 5′ end of the *(CAA)_4_ CAT-FLAG* mRNA−to block ribosomal recruitment at the cap-structure−inhibited expression of both proteins by more than 250-fold(**Figure 1B**; see 5′ hairpin, long exposure). This level of inhibition was determined by comparing the signals to those from 3-fold loading dilutions of CAT-FLAG proteins expressed from the parent construct. Another possibility is that a shorter mRNA species containing only AUG2 is generated by splicing of the primary transcript to remove sequences containing AUG1. The generation of such a monocistronic mRNA was considered unlikely as the 5′ leader lacks any predicted splice donor sites. Nevertheless, RT-PCR reactions were performed to test this possibility by using oligonucleotide primers located at the 5′ end of the mRNA and near the 3′ end of the coding region. These reactions produced a single cDNA band corresponding in size to the mRNA construct (**Figure 1C**; +RT, the band is indicated by an arrow labeled "S"). No smaller cDNA products were detected. The CAT-FLAG construct contains an intron in the coding sequence and a small amount of the unspliced mRNA was observed in the reactions (**Figure 1C**; +RT, the band is indicated by an arrow labeled "U"). These control experiments indicate that translation initiation in the *(CAA)_4_ CAT-FLAG* mRNA occurs from mRNAs that contain both AUG1 and AUG2.

The inhibitory effect of the 5′ hairpin structure on the expression of both CAT-FLAG proteins suggests that ribosomal recruitment was blocked by the hairpin structure and that translation of this mRNA is cap-dependent. To further investigate the ribosomal recruitment mechanism, we determined whether sequences upstream of AUG2, including the 5′ leader, AUG1, and sequences contained between the two AUG codons, could facilitate internal initiation of translation. These sequences were tested in the intercistronic region of a dicistronic mRNA encoding *Renilla* and *Photinus* luciferases. The results show that sequences from the CAT-FLAG construct did not drive second cistron expression above the levels observed for the negative control constructs, which are the parent RP construct (no insert) and the construct containing the *β-globin* 5′ leader (**Figure 1D**). These levels are ≈10 and 45-fold lower than the levels obtained from the Encephalomyocarditis virus (EMCV) and Poliovirus (PV) IRES constructs, respectively. This result indicates that sequences upstream of AUG2 in the *CAT-FLAG* mRNA do not facilitate internal initiation of translation. In addition, these results provide further evidence that the sequences upstream of AUG2 do not contain cryptic transcription start sites that might drive production of monocistronic (AUG2) mRNAs.

### Relative use of an AUG codon can be altered by an antisense oligonucleotide

**Figure 2:**
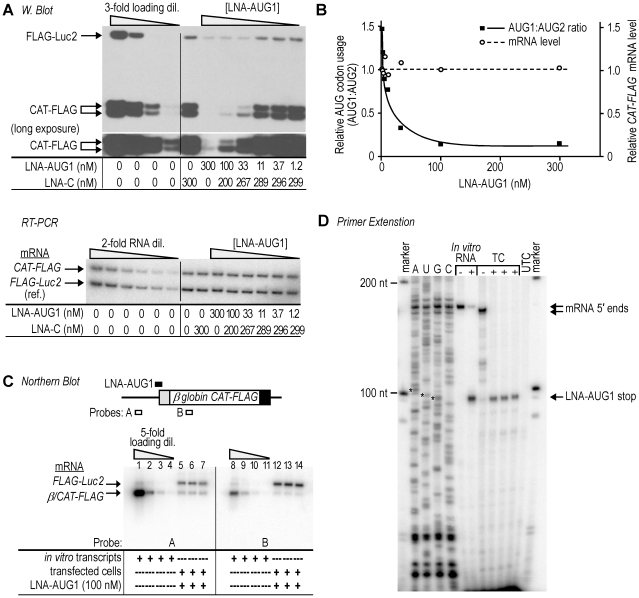
LNA-AUG1 modulates translation of the target mRNA. **A**. The top panel is a Western blot analysis of COS-7 cells transfected with plasmid constructs expressing *(CAA)_4_ CAT-FLAG* and *FLAG-Luc2* mRNAs, together with various amounts of LNA-AUG1 oligonucleotide. The lower panel is a semi-quantitative RT-PCR analysis of *(CAA)_4_ CAT-FLAG* mRNA expression in cells exposed to different amounts of LNA oligonucleotides. Duplex RT-PCR reactions were performed to analyze the levels of the control *FLAG-luc2* mRNA in each sample. **B**. Quantification of the effects of LNA-AUG1. An indication of relative AUG codon usage (left ordinate) is indicated by black squares and solid line and is obtained by normalizing the expression of the 31 kDa protein from AUG1 to that of the 28 kDa protein from AUG2. This ratio is plotted against concentration of LNA-AUG1 oligonucleotide. Relative expression of normalized *CAT-FLAG* mRNA levels (right ordinate; expressed relative to the sample with no LNA cotransfection) are indicated by open circles and dashed line. **C**. Target mRNA remains intact in cells cotransfected with LNA-AUG1. 1.0X *β-globin CAT-FLAG* mRNA from COS-7 cells cotransfected with LNA-AUG1 in independent triplicates was analyzed by Northern blot using probes that hybridize to regions upstream or downstream of the LNA-AUG1 target site. The diagram shows the relative positions of the probes. The control *FLAG-luc2* mRNA was detected by a subsequent hybridization using probes specific to this control mRNA. The five-fold dilutions were of an equivalent *in vitro* transcribed RNA. **D**. Primer extension analysis of RNA samples tested by Northern analysis using a primer that anneals 23-nucleotides downstream of AUG2. An *in vitro* transcribed RNA was included as a control for the position of primer extension inhibition by LNA-AUG1 binding. This RNA was incubated with (+) or without (-) LNA-AUG1. The sequencing ladder is from the corresponding plasmid; the marker is DNA. RNA samples from cells transfected with plasmid (TC; -), with plasmid and LNA-AUG1 (TC; +), and from untransfected cells (UTC) were analyzed in parallel. The positions of the mRNA 5′ ends and LNA stop sites are indicated by arrows. The position of AUG1 is indicated by asterisks in the sequencing ladder.

An LNA antisense oligonucleotide (LNA-AUG1) was designed to target AUG1 in the *(CAA)_4_ CAT-FLAG* mRNA. This oligonucleotide was cotransfected into cells along with plasmid constructs expressing the *(CAA)_4_ CAT-FLAG* mRNA and a cotransfection control mRNA (*FLAG-Luc2*). The LNA-AUG1 oligonucleotide was tested at different dilutions while keeping the total amount of oligonucleotide constant in each transfection reaction by using a non-specific isosequential LNA oligonucleotide (LNA-C) as filler. The results showed that expression of both the large and small CAT-FLAG proteins was differentially inhibited in a manner dependent on the amount of cotransfected LNA-AUG1 (**Figure 2A**, Western blot). Although translation from both AUG codons was decreased, translation from AUG1, which is targeted by LNA-AUG1, was decreased substantially more (>3-fold) than translation from AUG2. This decrease was most pronounced at oligonucleotide concentrations above 33 nM (**Figure 2B**). The relative expression levels of *CAT-FLAG* mRNAs were not significantly affected by cotransfection of cells with LNA-AUG1, as measured by semi-quantitative RT-PCR (**Figure 2A**, RT-PCR; and **2B**). This result suggests that the effect of the LNA-AUG1 oligonucleotide on the *(CAA)_4_ CAT-FLAG* mRNA is post-transcriptional and is not due to degradation of the target mRNA.

To determine whether the LNA-AUG1 oligonucleotide binds to its complement in the *(CAA)_4_ CAT-FLAG* mRNA, primer extension inhibition assays were performed using RNA extracted from COS-7 cells that were cotransfected with 100 nM LNA-AUG1. Toeprinting assays using *in vitro* transcripts incubated with increasing amounts of the LNA-AUG1 oligonucleotide indicated that LNA-AUG1 binds to the target sequence in LNA-transfected cells comparable to controls (**Figure S1A**). We did not detect any smaller primer extension products, suggesting that the LNA cotransfection did not induce transcription of shorter mRNAs.

To show that LNA-AUG1 does not induce cleavage at the target site in the mRNA, RNA was extracted from transfected COS-7 cells and analyzed by Northern blot hybridization using probes complementary to sequences located upstream or downstream of the oligonucleotide-binding site in the mRNA. We did not use the *(CAA)_4_ CAT-FLAG* mRNA for this analysis as the 5′ leader is too short for efficient hybridization; therefore experiments were performed using a similar mRNA that contains the longer *β-globin* 5′ leader. This mRNA, the *1.0X β-globin CAT-FLAG* mRNA, is appropriate for determining if LNA-AUG1 induces mRNA cleavage because this mRNA contains the same LNA-AUG1 binding site as the *(CAA)_4_ CAT-FLAG* mRNA. The Northern blot results were quantified using signals obtained from a serial dilution of equivalent *in vitro* transcripts, which were hybridized using the appropriate probes in parallel. The Northern analyses revealed a single band corresponding to the transiently expressed mRNA (**Figure 2C**) without detectable smaller species that might be suggestive of degradation and/or cleavage of the mRNA. In addition, no major differences were observed in *CAT-FLAG* mRNA levels, regardless of probe-hybridization position, i.e. upstream or downstream of AUG1, in the presence of the LNA1-AUG1 oligonucleotide. To illustrate, by comparing the intensities of the *CAT-FLAG* mRNAs in lanes 5–7 and 12–14 to those from the *in vitro* transcripts in lanes 1–3 and 8–10, respectively, it can be seen that for both probes, the mRNA intensities are slightly more intense than those obtained for the most dilute *in vitro* transcripts. These results indicate that the mRNA levels are equivalent upstream and downstream of the oligonucleotide-binding site. Similar results were obtained for Northern blot hybridizations performed in the absence of the LNA1-AUG1 oligonucleotide (data not shown). These results indicate that the LNA oligonucleotide did not induce mRNA cleavage. Primer extension analysis of the same RNA samples analyzed by Northern blotting confirms the binding of LNA-AUG1 to the target mRNAs (**Figure 2D**). All of these analyses strongly suggest that the LNA-AUG1 oligonucleotide affects translation by base pairing to the target mRNA−not by inducing production of an mRNA species lacking AUG1.

The primer extension results appear to reflect LNA-mRNA associations inside cells rather than binding that occurs after cell lysis. We determined this by adding a sufficient amount of DNA oligonucleotide complementary to LNA-AUG1 to cells prior to lysis in order to sequester unbound LNA oligonucleotides and prevent them from binding the target mRNA after cell lysis. Primer extension analysis revealed that essentially no detectable full-length product was observed in the presence of the highest amount of DNA oligonucleotide, when LNA-AUG1 was transfected into cells. However, full-length product was observed when LNA-AUG1 was added exogenously to cell lysates in the presence of the DNA oligonucleotide, suggesting that the DNA oligonucleotide could sequester unbound LNA-AUG1 (**Figure S1B**).

The LNA-AUG1 oligonucleotide preferentially decreased the expression from AUG1, but also decreased expression to some extent from AUG2. We therefore hypothesized that the inhibition at AUG2 may be a steric effect caused by binding of the 13-nt LNA oligonucleotide to the mRNA. To investigate this possibility, we performed experiments using a shorter (9-mer) LNA oligonucleotide to mask AUG1. The results showed that this oligonucleotide clearly inhibited translation from AUG1 (≈30%) while having only a very modest inhibitory effect on translation from AUG2 (**Figure S2**). This result supports the notion of a steric inhibitory effect of the 13-nt LNA oligonucleotide on translation from AUG2. The fact that the 9-nt oligonucleotide required a higher concentration to affect translation than the 13-nt oligonucleotide may be due to the decreased binding stability of the shorter LNA oligonucleotide.

### Relative use of two AUG codons can be altered by varying the length of 5′ leader

**Figure 3:**
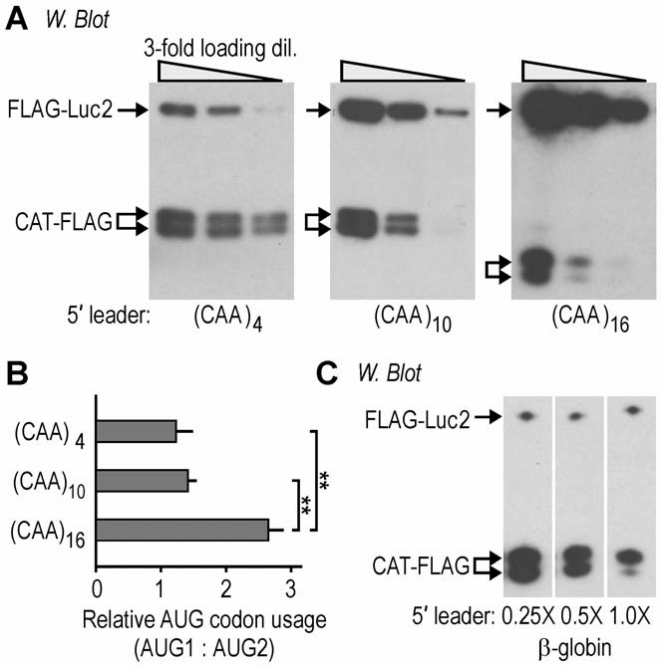
Relative translation at two AUG codons is altered by the length of 5′ leader. **A**. Western blot analysis of the two CAT-FLAG proteins expressed from *(CAA)_4_, (CAA)_10_*, and *(CAA)_16_ CAT-FLAG* mRNAs in COS-7 cells that are also transiently expressing the control *FLAG-Luc2* mRNA. **B**. The histogram shows the expression of CAT-FLAG protein initiating at AUG1 relative to the expression initiating at AUG2. At least three independent experiments were performed for final quantification of the immunoblot, with error bars indicating standard deviations. The mRNA containing 16 tandem repeats of CAA shows a significantly higher AUG1:AUG2 ratio than those with 4 or 10 tandem repeats (** one-sided t-test: p<0.01). (**C**) Immunoblot analysis of the two CAT-proteins expressed from *0.25X, 0.5X and 1.0X β-globin CAT-FLAG* mRNAs in COS-7 cells.

**Figure 4:**
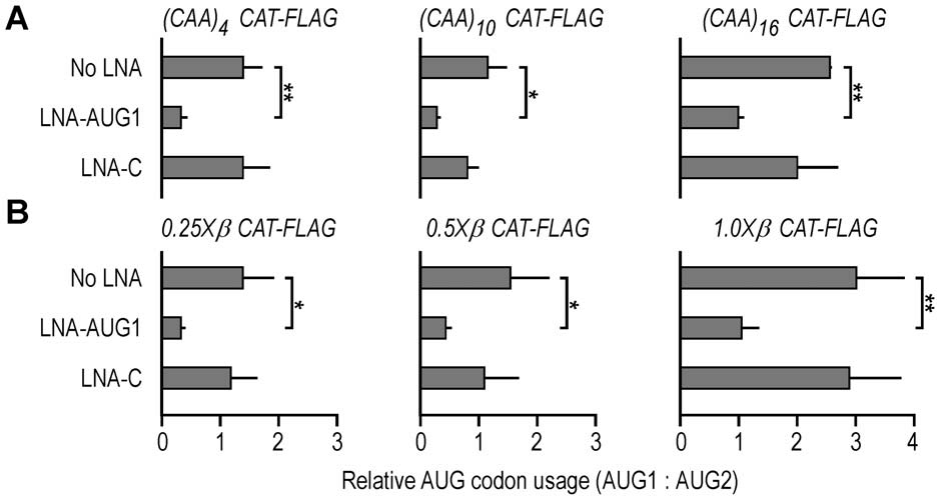
Effect of LNA-AUG1 is independent of different 5′ leader lengths. **A**. CAT-FLAG protein expression from AUG1 normalized with that from AUG2 in *(CAA)_n_ CAT-FLAG* mRNAs. COS-7 cells were transfected with plasmid constructs that express *(CAA)_4_*, *(CAA)_10_*, or *(CAA)_16_ CAT-FLAG* mRNAs, along with a control plasmid that expresses the control *FLAG-Luc2* mRNA. LNA oligonucleotides (100 nM) were cotransfected into COS-7 cells as indicated. Expression levels of the two CAT-FLAG proteins were analyzed as in Figure 2. **B**. CAT-FLAG protein expression from AUG1 normalized with that from AUG2 in COS-7 cells transfected with a plasmid construct that expresses *0.25X, 0.5X, or 1.0X β-globin CAT-FLAG* mRNA. At least three independent experiments were performed for final quantification of the immunoblot with error bars indicating standard deviations. For these experiments, cotransfection of cells with LNA-AUG1 resulted in significant differences in relative AUG codon usage (one-sided t-test: * p<0.05; ** p<0.01); no significant change in relative AUG codon usage was observed in cells cotransfected with LNA-C (p>0.05).

As noted above in Figure 1B, the robust use of AUG2 was observed despite the optimal nucleotide context of AUG1. The 5′ leaders in this study are sufficiently long to be able to circumvent leaky scanning, which suggests that for 5′ leaders shorter than 10-nucleotides, ribosomal subunits are more likely to bypass the first AUG codon and initiate translation at a downstream codon [12]. To further evaluate this possibility, we tested synthetic *CAT-FLAG* mRNAs with longer 5′ leaders. These mRNAs, with 4, 10, or 16 tandem repeats of the CAA trinucleotide were tested in transiently transfected COS-7 cells. The results showed that both proteins were expressed approximately equally from the constructs containing 4 and 10 CAA repeats in their 5′ leaders (**Figure 3A, B**); however, for the construct with 16 CAA repeats in its 5′ leader, the protein initiating at AUG1 was expressed ≈2.5-fold higher than that initiating at AUG2. Similar results were observed using 5′ leader sequences based on the *β-globin* 5′ leader, which contains more secondary structure than CAA repeats [23]. These mRNAs contained the full-length *β-globin* 5′ leader (1.0X) or shorter segments (0.5X and 0.25X; **Figure 3C**; [4]). As with the (CAA)_16_ 5′ leader, the mRNA containing the *β-globin* 5′ leader (1.0X), which has approximately equal leader length, expressed the protein from AUG1 at a level ≈3-fold higher than the protein from AUG2. Likewise, the constructs with the shorter 5′ leaders (0.5X and 0.25X) expressed the two proteins at roughly equal levels, similar to the two shorter CAA constructs ((CAA)_4_ and (CAA_10_)). Control experiments indicated that translation from AUG2 was not due to cryptic induction of either transcription or IRES activity by the *β-globin* sequences (**Figure 1D**). Inasmuch as these results were obtained using two different 5′ leader sequences, they indicate that the length of the 5′ leader itself is a variable affecting the relative usage of AUG codons in mRNAs.

### Antisense oligonucleotide effects are independent of 5′ leader length

Constructs expressing mRNAs containing 5′ leaders with different numbers of CAA repeats or lengths of *ß-globin* 5′ leader sequences were cotransfected into COS-7 cells with various concentrations of the LNA-AUG1 oligonucleotide. At 100 nM, the ratio of AUG1:AUG2 usage was decreased by ≈2–3 fold for all constructs (**Figure 4A, B**), similar to the effect observed in *CAT-FLAG* mRNA containing the *(CAA)_4_* 5′ leader (**Figure 2A, B**). The ratios were not further altered when cells were cotransfected with 300 nM oligonucleotide, and could not be accounted for by alterations in mRNA levels (data not shown). Moreover, the relative effects of 5′ leader length were retained even in the presence of the LNA-AUG1 oligonucleotide. The ratios were not significantly altered when cells were co-transfected with LNA-C.

### Effects of oligonucleotides and 5′ leader length confirmed by RNA transfection

The preceding experiments expressed various mRNAs from plasmids transfected into cells and included numerous control experiments to rule out possible artifacts including alternative splicing and cryptic transcription. To provide an additional control for these studies and eliminate the possibility of plasmid-specific artifacts, we repeated key experiments using *in vitro* transcribed mRNAs transfected into COS-7 cells. A time course of protein expression showed similar expression of both CAT-FLAG proteins from the *(CAA)_4_ CAT-FLAG* mRNA (**Figure S3A**). In the presence of LNA-AUG1, there is an inhibition of translation from AUG1 at 4 hours post transfection. In addition, the effects of 5′ leader length observed in the plasmid transfection experiments were also seen in the RNA transfections (**Figure S3B**).

### The ratio of AUG codon usage was unaffected by oligonucleotides binding in 5′ leader

**Figure 5:**
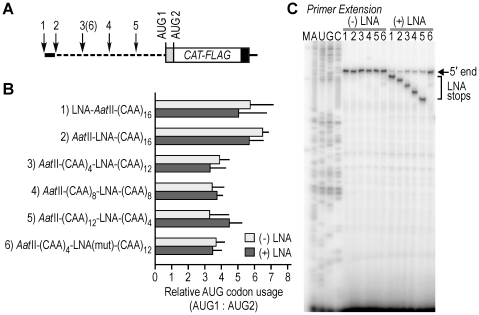
Ratio of AUG codon usage is affected by nucleotide composition of the 5′ leader. **A**. Schematic representation of *CAT-FLAG* mRNAs. The arrows (numbered 1-5) indicate the positions of individual LNA target sequences in the 5′ leaders of different constructs. Arrow 6 indicates the position of a mutated target sequence. The thick black bar at the 5′ end of the mRNA represents an *Aat*II sequence; dashes in the 5′ leader represent CAA repeats. **B**. Relative expression of CAT-FLAG proteins. Expression from AUG1 is normalized to that from AUG2 in COS-7 cells that were transfected with *in vitro* transcribed capped and poly(A)-tailed mRNAs with 5′ leader sequences as depicted in (A). Transfections were performed in the presence (+; dark grey bar) and absence (-; light grey bar) of LNA oligonucleotide. The cells were harvested 5 hours post transfection. Three independent experiments were performed for final quantification of immunoblots with error bars indicating standard deviations. Cotransfection with LNA oligonucleotide did not significantly alter relative AUG usage (two-sided t-test). One-way ANOVA analysis of relative AUG codon usage in constructs 3–6 ((-)LNA) did not show significant differences. However, there were some significant differences between constructs 1 or 2 ((-)LNA) and various other constructs. One-sided t-tests of construct 1 compared to constructs 3–6 ((-)LNA) yielded p-values of 0.06, 0.04, 0.04, and 0.06, respectively. One-sided t-tests of construct 2 compared to constructs 3–6 ((-)LNA) yielded p-values of <0.01, <0.01, 0.02, and <0.01, respectively. **C**. Primer extension inhibition analysis on target mRNAs bound with LNA oligonucleotide. Total RNA extracted from cells 5 h post transfection was analyzed by primer extension to confirm the positions of LNA binding to target mRNAs, using a primer that anneals 67-nucleotides downstream of AUG2. The extension products were resolved using 6% denaturing PAGE along with a DNA size marker (M) and sequencing ladder from the plasmid with LNA-target site-*Aat*II-(CAA)_16_. Primer extension reactions of RNA samples from COS-7 cells cotransfected with (+) or without (-) LNA oligonucleotide were compared in parallel. The results are representative of three experiments performed independently.

To determine whether the ratio of utilization of two AUG codons is altered by binding of an antisense LNA oligonucleotide to various locations in a 5′ leader other than that of AUG1, we generated a series of mRNA constructs with isosequential 5′ leaders by inserting an LNA-target sequence into different parts of the 5′ leader of the *(CAA)_16_ CAT-FLAG* mRNA (**Figure 5A**). The binding site used in this study is complementary to the LNA-C oligonucleotide, which was used as a control in our earlier studies. This set of studies was performed using RNA transfections as in Figure S3, because we found that it was easier to control the mRNA:LNA oligonucleotide ratio in cells using this approach, compared to plasmid cotransfections, which express the various recombinant mRNAs at different levels. For these studies, COS-7 cells were transfected with the various *CAT-FLAG* mRNAs and protein expression was quantified. In the absence of the LNA oligonucleotide, AUG1 is preferentially used in all of the constructs, and the AUG1:AUG2 ratio is higher when the LNA binding site is located at or near the 5′-end of the transcript (**Figure 5B**, see constructs 1 and 2). This result suggests that the sequence composition of the 5′ leader may be another variable that can influence the relative usage of AUG codons in an mRNA, and which may affect ribosomal interactions with AUG codons by other means (see Discussion).

When the transcripts were bound with the LNA-C oligonucleotide, the ratios of AUG1:AUG2 codon usage obtained from the various constructs were largely unaltered (**Figure 5B**). However, the levels of both proteins were significantly lower than for an mRNA containing a mutated LNA-C binding site (*AatII-(CAA)_4_-LNA(mut)-(CAA)_12_;* see **Figure S4**). Binding of the LNA-C oligonucleotide to specific sites was confirmed by primer extension inhibition on RNA extracted from transfected cells (**Figure 5C**). Control experiments indicated that these various 5′ leaders (including sequences upstream of AUG2) do not appear to have IRES activity when placed in the intercistronic region of a dicistronic mRNA encoding *Renilla* and *Photinus* luciferases (**Figure S5**). None of these sequences tested yielded *Photinus* luciferase activities (encoded in the second cistron) higher than the negative control (RP; no insert) construct. These results indicate that binding of an antisense oligonucleotide in the 5′ leader of an mRNA does not necessarily affect the ratio of utilization of two AUG codons, but does have a general inhibitory effect.

### AUG codon usage affected by exon-junction complex deposition

**Figure 6:**
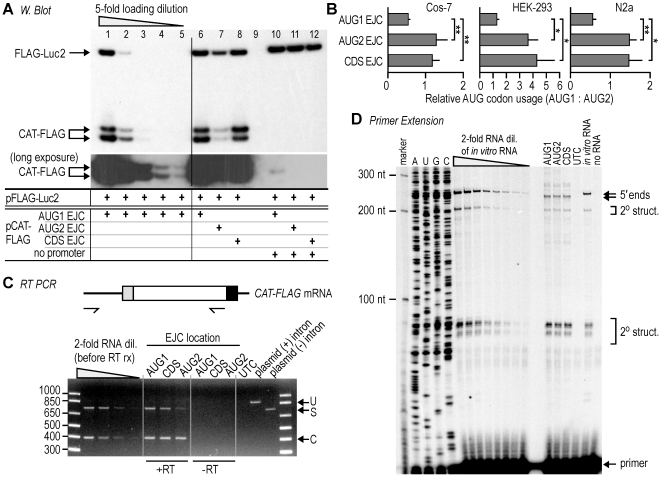
Targeting EJC to AUG1 affects relative use of two AUG codons. **A**. Immunoblot analysis of CAT-FLAG protein expression in COS-7 cells from constructs that contain an intron at one of three positions, such that upon splicing an EJC is deposited on AUG1, AUG2 or the CAT coding region. COS-7 cells were cotransfected with CAT-FLAG and the control FLAG-Luc2 plasmids. Equivalent constructs lacking SV40 promoter/enhancer sequences (no promoter) were analyzed in parallel. Five-fold dilutions are of a sample with the EJC on AUG1. A longer film exposure of the blot is shown for the CAT-FLAG protein. **B**. Relative expression of CAT-FLAG proteins in COS-7, HEK-293 and N2A cells was quantified from immunoblots and shown as an AUG1:AUG2 ratio for each construct. At least three independent experiments were performed for final quantification of the immunoblot with error bars indicating standard deviations. Asterisks indicate statistically significant differences (one-sided t-test: *p<0.05; ** p<0.01). **C**. RT-PCR analysis of mRNA constructs. The size control for the PCR products of unspliced (U) or correctly spliced (S) *CAT-FLAG* mRNAs was amplified from plasmids that contain or lack the intron sequence, respectively, using the same duplex PCR conditions in parallel. The arrow labeled C indicates the RT-PCR product from the control *FLAG-Luc2* mRNA. The two-fold dilution of RNA samples was reverse-transcribed prior to PCR amplification. **D**. Primer extension analysis of mRNA expressed from each construct in COS-7 cells. The RNA sample from each construct was analyzed for a primer extension profile, using a primer that annealed 67-nucleotides downstream of AUG2. To control for the presence of secondary structures that may inhibit the primer extension, an equivalent RNA lacking an intron was transcribed *in vitro* from the T7 RNA polymerase promoter, and included in the analysis. Controls for nonspecific stops of the primer extension are RNAs extracted from untransfected cells (UTC) and a blank reaction (no RNA). The left lanes contain a sequencing ladder that is derived from the equivalent plasmid, which lacks an intron, and the DNA size marker (marker). The position of the mRNA 5′ ends, secondary structures (2° struct.), and free primer are indicated to the right. The results are representative of at least three independent experiments.

To control for possible unanticipated effects of LNA oligonucleotides on the ratio of AUG1:AUG2 codon use, we used a different obstacle to mask AUG1 in a synthetic mRNA. The EJC appeared to be attractive for this application as this protein complex is deposited on mRNAs 20–24 nucleotides upstream of exon-exon junctions during or after splicing [17]. To explore whether an EJC deposited on an AUG codon may negatively affect initiation at this site, we prepared three sister constructs that contain an intron at different locations, such that an EJC is deposited on AUG1, AUG2, or in the *CAT* coding region. We expected that EJCs on AUG2 and in the coding region would be removed by any ribosomes that initiate translation at AUG1, and that EJCs at these sites should not affect AUG choice beyond the first round of translation initiation, and thus would not affect the relative usage of AUG codons. Although the various intron positions generate constructs that differ at the DNA and pre-mRNA levels, the primary sequences of the spliced mRNAs are identical. The results revealed that cells transfected with the construct targeting the EJC to AUG1 expressed more protein from AUG2 than from AUG1 (**Figure 6A, B**). By contrast, cells transfected with the constructs targeting the EJC to AUG2 or the coding region expressed more protein from AUG1 than from AUG2. This result is consistent with the notion that EJCs are removed from coding regions by ribosomes during translation [24]. Similar results were obtained when the various constructs were tested in various cells including human embryo kidney HEK-293 and mouse neuroblastoma N2a cells (**Figure 6B**). It is interesting to note that in HEK293 cells, translation was more strongly biased to AUG1 than in COS-7 or N2a cells (note differences in the abscissae of Figure 6B), and that EJC deposition on AUG1 has a more pronounced effect in HEK293 cells than in the other cell lines tested. We consistently observed lower expression from the construct in which the EJC is targeted to AUG2 (≈25%). This lower expression is explained by lower mRNA levels (**Figure 6C**). Nevertheless, the use of the two AUG codons in this construct was similar to that observed from the construct in which the EJC is deposited in the coding region.

The sequences preceding AUG2 are identical to those tested in **Figure 1D** and do not generate cryptic transcripts or have IRES activity. In addition, RT-PCR reactions were performed using a primer pair that anneals to the 5′ end of the mRNA and to the 3′ end of coding region. The results showed that primer specific amplification of *CAT-FLAG* cDNA was detected only in reactions containing reverse transcriptase (+RT); not in reactions for which it was omitted (-RT) or in RNA samples from untransfected cells (UTC; **Figure 6C**). Moreover, no unexpected RT-PCR products were detected, suggesting that no alternative splicing occurred that could have generated altered AUG codon-usage ratios. Moreover, primer extension reactions on RNA extracted from cells transfected with the three constructs yielded patterns that were identical to each other and virtually identical to those obtained from an *in vitro* transcript (**Figure 6D**). These control experiments indicate that the three EJC constructs give rise to mature transcripts that are identical in primary sequence and differ only in the position of EJC deposition. These data support the conclusion that the presence of an EJC on an AUG codon can diminish its use as an initiation codon.

## Discussion

In this study, we investigated AUG codon accessibility as a variable affecting translation initiation in mammalian cells. These studies were performed using synthetic reporter mRNA constructs that contain two in-frame AUG codons, both of which reside in nucleotide contexts that are considered excellent for translation initiation. The synthetic mRNAs used in these studies were demonstrated to recruit ribosomes by a cap-dependent mechanism and to initiate translation efficiently from both AUG codons. Control experiments ruled out the possibility that initiation from AUG2 occurred from monocistronic mRNAs lacking AUG1. The results of this study showed that it is possible to alter the ratio of usage of two AUG codons in an mRNA by various means. These include reducing the accessibility of the first AUG codon by masking it with an LNA antisense oligonucleotide or by depositing an EJC on this codon.

The results of the present study are consistent with those of previous experiments in yeast carried out *in vitro* using a *CAT* reporter mRNA similar to our construct [25]. This study reported that binding of 2′-O-allyloligonucleotides to either of two in-frame AUG codons specifically inhibited translation predominantly from the target site and resulted in highly skewed ratios of protein expression (see supplementary figure 2 in reference [25]). In addition, our studies demonstrate that it is possible to alter the ratio of AUG codon usage not only by altering the length of the 5′ leader, but also by changing the nucleotide composition of the 5′ leader, or expressing the RNA in different mammalian cell lines.

In one set of experiments, the ratio of usage of the two AUG codons was altered by reducing the accessibility of AUG1 and cotransfecting cells with an LNA antisense oligonucleotide targeted to AUG1 (LNA-AUG1). LNA antisense oligonucleotides tend to be more effective than other types of antisense oligonucleotides [26]. In our study, the LNA-AUG1 oligonucleotide inhibited translation from AUG1 in a dosage dependent manner (**Figure 2**) and was shown to form a duplex at the intended location (**Figure S1**). Importantly, the relative expression from AUG2 was increased by blocking AUG1 (**Figures 2** and **4**). Expression from both AUG codons was reduced, indicating that both translation products were derived from mRNA templates that contain both AUG codons. This notion is supported by numerous control experiments including mRNA transfections, which showed that translation from AUG2 could not be accounted for by cryptic promoter activity, internal initiation of translation, or mRNA splicing. The alteration in the ratio of AUG codon utilization appears to be independent of the inhibition of expression from AUG2 as experiments performed using a shorter LNA oligonucleotide (9- vs 13-nucleotides) yielded a similar change in ratio without significantly inhibiting expression from AUG2. In addition, an LNA oligonucleotide targeted to various sites in the 5′ leader reduced expression from both AUG codons without affecting the ratio of utilization. These results suggest that binding of an LNA oligonucleotide to the mRNA also has a general negative effect. This effect may be steric, e.g., the binding may inhibit ribosomal recruitment or reduce the flexibility of the 5′ leader. Alternatively, the binding may trigger the formation of translationally repressed RNPs.

In a second set of studies, the accessibility of an AUG codon was reduced by using an EJC to mask it (**Figure 6A**). Inasmuch as EJCs bind to mRNAs via protein interactions that are sequence independent, they provided a completely different type of obstacle than LNA oligonucleotides. We were able to target an EJC to various sites in the mRNA by inserting an intron 19–23 nucleotides downstream of these sites. This approach yielded mature mRNAs with identical primary sequences, as indicated by the lengths of the RT-PCR products and the primer extension profiles (**Figure 6C, D**). Thus, the resulting differences in AUG codon usage are consistent with the sites of EJC deposition. The ratio of utilization of the two AUG codons was altered to favor AUG2 when the EJC was targeted to AUG1. An EJC targeted to AUG2 or to the coding region had no effect on the ratio. This result was expected, as an EJC on AUG2 or in the coding region should be removed by ribosomes initiating translation at AUG1. These results are comparable to those obtained with the LNA oligonucleotides; however, in contrast to the LNA oligonucleotide experiments, the observed ratio change was not accompanied by decreased translation from AUG2 (compare lanes 6 and 8 in **Figure 6A**). These results suggest that an EJC deposited on AUG1 decreases its utilization. However, we were unable to obtain biochemical confirmation of an EJC on AUG1. One possibility is that ribosomes in the elongation phase remove EJCs from both 5′ leader and coding regions, but with different kinetics for each region. For example, it is generally thought that EJCs in coding regions are stripped off with ribosomal passage [24], [27]. However, EJCs in the 5′ leader are not necessarily removed prior to the first initiation event or with the first round of elongation, and removal from 5′ leaders may depend on where they are located relative to the initiation codon. This hypothesis is supported by various observations in the literature: 1) removal of EJCs from coding regions appears to require translation as they are not removed when translation is blocked by a stem-loop structure upstream of the initiation codon [24] or from a fully processed mRNA lacking ORFs [28]; 2) the latter example suggests that EJCs are not removed by preinitiation events, as an mRNA without an ORF is essentially equivalent to a 5′ leader; and 3) the disassembly of EJCs in coding regions involves a protein (PYM) that is associated with ribosomes and interacts with components of the EJC during translation [29]. The hypothesis that ribosomes remove EJCs with different kinetics from coding and 5′ leader regions provides a plausible explanation for why the EJC targeted to AUG1 in our studies distorted the ratio of AUG codon utilization but did not completely block translation initiation from AUG1. This hypothesis also suggests why it may be difficult to isolate mRNA complexes with an EJC on AUG1.

It is known that EJCs can function to promote translation [30], [31] and trigger efficient nonsense-mediated decay (NMD) when located more than 26–35 nucleotides downstream of a stop codon [27]. The present study raises the possibility that EJC deposition may also restrict where ribosomes initiate translation, at least for the first round of translation. Potential candidates for this type of regulation include human zinc finger protein 36 (Zfp36), thioredoxin, and signal recognition particle 14-kDa (SRP14). Each of these mRNAs has an intron located downstream of the initiation codon that would result in the deposition of an EJC on the initiation codon upon splicing. In addition to the possibility that EJC deposition may affect where translation initiates, the ability of ribosomes to initiate translation efficiently at more than one initiation codon has other implications for NMD. For example, some mRNAs may escape NMD if translation initiates at an alternative initiation codon and the ORF terminates less than 35-nucleotides upstream of an EJC in the 3′ UTR, or downstream of this EJC. By contrast, some alternative initiation events may trigger NMD if the ORF terminates more than 26 nucleotides upstream of an EJC. The present work provides a testable hypothesis for mRNAs that do not appear to follow the EJC-directed boundary rule [32], and that cannot be explained by reinitiation, which is able to inhibit NMD [33].

In addition to showing that the ratio of usage of two AUG codons could be altered by masking the first AUG codon by different means, our studies showed that the ratio of AUG codon usage could be altered by varying the length of the 5′ leader, the nucleotide composition of the 5′ leader, and the cell line. The effect of 5′ leader length is consistent with our earlier studies [4], and with other studies that have reported effects on translation efficiency and AUG codon choice (e.g. see references [12], [34]). However, it is not possible generally to deduce the effects of specific 5′ leader lengths on translation, as different nucleotide compositions may generate different secondary structures, or contain binding sites for proteins or other nucleic acids. The idea that the distance between a ribosomal recruitment site and an AUG codon can affect translation initiation also seems to apply to translation in bacteria that involves ribosomal recruitment at a Shine-Dalgarno sequence [35], [36], as well as to some animal viruses and plant viruses [22], [37], [38], [39]. The fact that we found differences in the relative usage of two AUG codons in different cell lines (**Figure 6B**) raises the possibility that a cellular factor or factors may differentially affect AUG codon selection, either by affecting the mRNA or the ribosome itself.

Our data demonstrate that multiple protein isoforms can be expressed from a single mRNA and the levels of these isoforms can be regulated by the length of the 5′ leader as well as by factors affecting the relative accessibilities of various initiation codons. While these results appear to be consistent with an hypothesized nonlinear mechanism of ribosomal movement from the cap to the AUG codon [4], [40], the experiments were not specifically designed to address the mechanism of ribosomal movement during translation initiation, a process that requires further study.

The results of our study imply that there is flexibility inherent in the selection of translation initiation sites, and that this process is modifiable. We suspect that the ability of numerous variables to alter the ratio of usage of the two AUG codons in an mRNA reflects the complexity of translation initiation. We expect that as we understand the variables affecting translation initiation more fully, our ability to predict where translation initiates, and how efficiently, will improve. A question that arises is whether the translation of some natural mRNAs is affected by mechanisms that mask authentic or alternative initiation codons. Such factors may include the EJC, which was used in this study and which was discussed above. Other possible masking mechanisms include RNA secondary structures, which can mask AUG codons within helices and may restrict their use. Alternatively, masking may occur via *trans*-factors, including RNA binding proteins, or complementary RNAs. Candidate RNAs include miRNAs, which are short, and in some cases highly abundant. Indeed, a search of miRNA seed sequences reveals several miRNAs that can potentially recognize AUG codons in various nucleotide contexts, raising the possibility that particular miRNAs affect the translation of some mRNAs by masking start sites. In addition to factors that affect the relative accessibility of an initiation codon, we anticipate that the selection of initiation codons can be affected by factors that alter the flexibility of the 5′ leader. It will be interesting to determine whether such mechanisms underlie the unusual translation initiation properties of mRNAs such as *BACE1* [41], [42]. This mRNA is cap-dependent and initiates translation at the fifth AUG codon; however, depending on the cell line and experimental conditions, the upstream AUG codons are either completely bypassed, or there is some initiation, mostly at AUG2. It may also be interesting to investigate the regulated expression of particular protein variants that are associated with different activities. For example, the CCAAT/enhancer–binding protein *β* [43] and the G-protein signaling-2 protein [44] both have several isoforms that vary at their amino termini and are associated with distinctive biological activities. Finally, the results of this study corroborate numerous other studies indicating that a substantial fraction of the proteome may consist of multiple peptides and proteins that are encoded by individual mRNAs, both from the same and different reading frames [45], [46].

## Supporting Information

Figure S1 **Inhibition of primer extension by specific LNA-binding to mRNA.** (**A**) Primer extension analysis of *(CAA)_4_ CAT-FLAG* mRNAs expressed in COS-7 cells. Primer extension was performed on duplicate RNA samples extracted from untranfected cells (UTC), cells transfected with plasmid expressing *(CAA)_4_ CAT-FLAG* mRNA (TC) or cells cotransfected with this plasmid and LNA-AUG1 or LNA-C. Primer extension reactions used ^32^P-labeled primers that anneal 23-nucleotides downstream of AUG2. *In vitro* transcribed RNAs (0.01 µM) incubated with different concentrations of LNA-AUG1 (10, 1, 0.1 and 0.01 µM) were analyzed in parallel (indicated by triangle, right 4 lanes). The positions of the mRNA 5′ ends and LNA stop sites are indicated by arrows. The left lanes contain a sequencing ladder that is derived from the corresponding plasmid, and the DNA size marker (marker). (**B**) Primer extension analysis on *(CAA)_4_ CAT-FLAG* mRNAs from COS-7 cells supplemented with different amounts of anti-LNA-AUG1 DNA oligonucleotide (α-LNA DNA oligo; 0 to 16 nmol, indicated by black triangles), prior to lysis. The nucleotide sequence of the DNA oligonucleotide is identical to the LNA-AUG1 target sequence in the mRNA, allowing competitive sequestration of the free LNA oligonucleotide. LNA-AUG1 was either cotransfected at 100 nM with the plasmid expressing *(CAA)_4_ CAT-FLAG* mRNA (left lanes), or added to the cell lysate (16 pmol; right lanes). The primer extension was performed using a primer that anneals 23-nucleotides downstream of AUG2. In parallel, primer extension reactions were performed on the five-fold dilutions of the corresponding *in vitro* transcript (0.08 to 50 fmol; grey triangle).
(TIF)

Figure S2 **9-nt LNA-AUG1 modulates translation of the target mRNA with minimal inhibitory effect on AUG2.** (**A**) A 9-mer LNA oligonucleotide that targets AUG1 was cotransfected into COS-7 cells with plasmids expressing *(CAA)_4_ CAT-FLAG* and *FLAG-Luc2* mRNAs. Two different concentrations of the LNA oligonucleotide were tested: 1 µM and 3 µM. (**B**) The relative protein expression (AUG1:AUG2) was quantified as in Figure 2 and plotted as a histogram, with error bars indicating standard deviations. Three independent experiments were performed to calculate the relative protein expression. Asterisks indicate statistically significant differences (** one-sided t-test: p<0.01).
(TIF)

Figure S3 **RNA transfections in COS-7 cells.** (**A**) Time course of COS-7 cells transfected with *in vitro* transcribed *0.25X β-globin CAT-FLAG* and *FLAG-luc2* mRNAs. mRNAs were 5′-capped and poly(A)_70_-tailed *in vitro* transcripts (1 pmol each). Growth media was exchanged 1 hour post transfection and cells harvested at times indicated. An equal volume of cellular lysate was loaded in each lane of SDS-PAGE, along with 8-fold serial dilutions of the lysate. The expressed proteins were detected by anti-FLAG monoclonal antibody. Three different film exposures are shown. (**B**) Effects of 5′ leader length on AUG-codon usage in *in vitro* transcribed mRNAs. COS-7 cells were transfected with 5′-capped and poly(A)_70_-tailed *in vitro* transcripts (*0.25X, 0.5X or 1.0X β-globin CAT-FLAG* and *FLAG-Luc2*; 1 pmol each). Cells were harvested 5 hours post transfection. Three-fold dilutions of cell lysates were analyzed by Western blot using anti-FLAG monoclonal antibody.
(TIF)

Figure S4 **Effects of binding an LNA-oligonucleotide to different sites in the 5′ leader.** (**A**) Schematic representation of constructs. The arrows (numbered 1-5) indicate the positions of individual LNA target sequences in the 5′ leaders of different constructs. Arrow 6 indicates the position of a mutated LNA target sequence. The thick black bar at the 5′ end of the mRNA represents an *Aat*II sequence; the dashes in the 5′ leader represent CAA tri-nucleotide sequences. (**B**) COS-7 cells were transfected with 1 pmol each of 5′-capped and poly(A)_70_-tailed *in vitro* transcripts of *FLAG-Luc2* and *(CAA)_16_ CAT-FLAG* mRNA variants that differ in the location of an LNA target site in the 5′ leader. 10 pmol of LNA-C, which is complementary to the LNA target sites in the 5′ leaders, was preincubated with the mRNA solutions before transfection. Total protein expression from AUG1 and AUG2 was quantified from Western blots by using the FLAG-Luc2 protein as a reference and plotted in a histogram, as a fraction of CAT-FLAG expression from an equivalent mRNA transfection, but without preincubation with LNA-C. Three independent experiments were performed for final quantification of the immunoblot with error bars indicating standard deviations. Asterisks indicate statistically significant differences with construct 6 (one-sided t-test: ** p<0.01).
(TIF)

Figure S5 **Dicistronic mRNA analysis of 5′ leaders with LNA binding sites at various locations.** Sequences upstream of AUG2 in the *(CAA)_16_ CAT-FLAG* mRNA variants were tested in the intercistronic region of the *Renilla*/*Photinus* dual luciferase dicistronic mRNA for IRES activity as in Figure 1D. Vector sequences in the parent (RP) construct were used as a negative control. The results were plotted in a bar graph relative to the *Renilla* (rLuc) and *Photinus* luciferase (pLuc) activities from RP, which are individually defined as 1. Three independent experiments were performed for final quantification with error bars indicating standard deviations.
(TIF)

Methods S1 (DOC)
