# Supplementary figures and images for: Determinants of Initiation Codon Selection during Translation in Mammalian Cells
Source: PLoS One. 2010 Nov 24;5(11):e15057. doi: 10.1371/journal.pone.0015057 (PMC2991327; doi:10.1371/journal.pone.0015057)

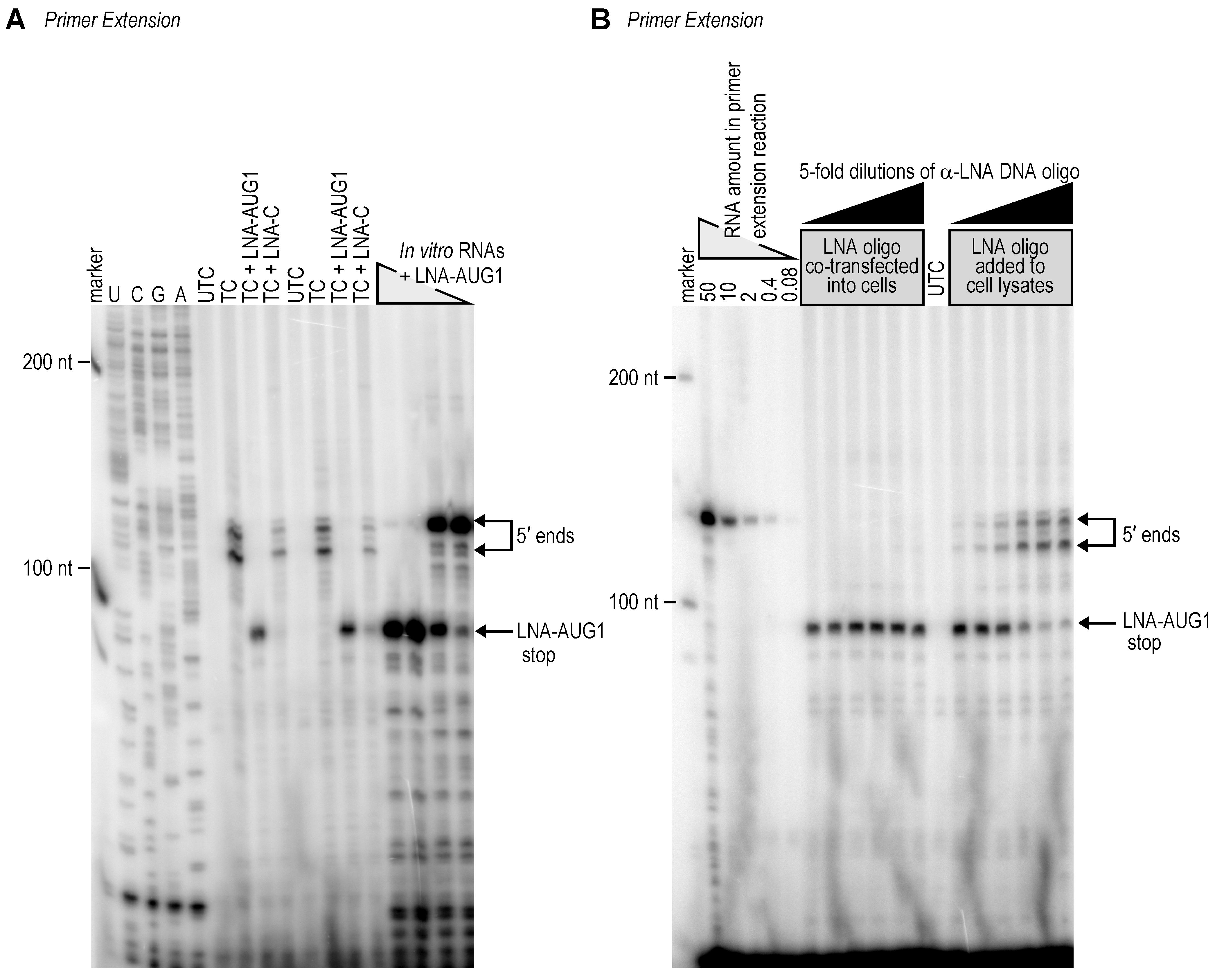

Supplement: Figure S1 — Inhibition of primer extension by specific LNA-binding to mRNA. (A) Primer extension analysis of (CAA)4 CAT-FLAG mRNAs expressed in COS-7 cells. Primer extension was performed on duplicate RNA samples extracted from untranfected cells (UTC), cells transfected with plasmid expressing (CAA)4 CAT-FLAG mRNA (TC) or cells cotransfected with this plasmid and LNA-AUG1 or LNA-C. Primer extension reactions used 32P-labeled primers that anneal 23-nucleotides downstream of AUG2. In vitro transcribed RNAs (0.01 µM) incubated with different concentrations of LNA-AUG1 (10, 1, 0.1 and 0.01 µM) were analyzed in parallel (indicated by triangle, right 4 lanes). The positions of the mRNA 5′ ends and LNA stop sites are indicated by arrows. The left lanes contain a sequencing ladder that is derived from the corresponding plasmid, and the DNA size marker (marker). (B) Primer extension analysis on (CAA)4 CAT-FLAG mRNAs from COS-7 cells supplemented with different amounts of anti-LNA-AUG1 DNA oligonucleotide (α-LNA DNA oligo; 0 to 16 nmol, indicated by black triangles), prior to lysis. The nucleotide sequence of the DNA oligonucleotide is identical to the LNA-AUG1 target sequence in the mRNA, allowing competitive sequestration of the free LNA oligonucleotide. LNA-AUG1 was either cotransfected at 100 nM with the plasmid expressing (CAA)4 CAT-FLAG mRNA (left lanes), or added to the cell lysate (16 pmol; right lanes). The primer extension was performed using a primer that anneals 23-nucleotides downstream of AUG2. In parallel, primer extension reactions were performed on the five-fold dilutions of the corresponding in vitro transcript (0.08 to 50 fmol; grey triangle). (TIF) [file pone.0015057.s001.tif]

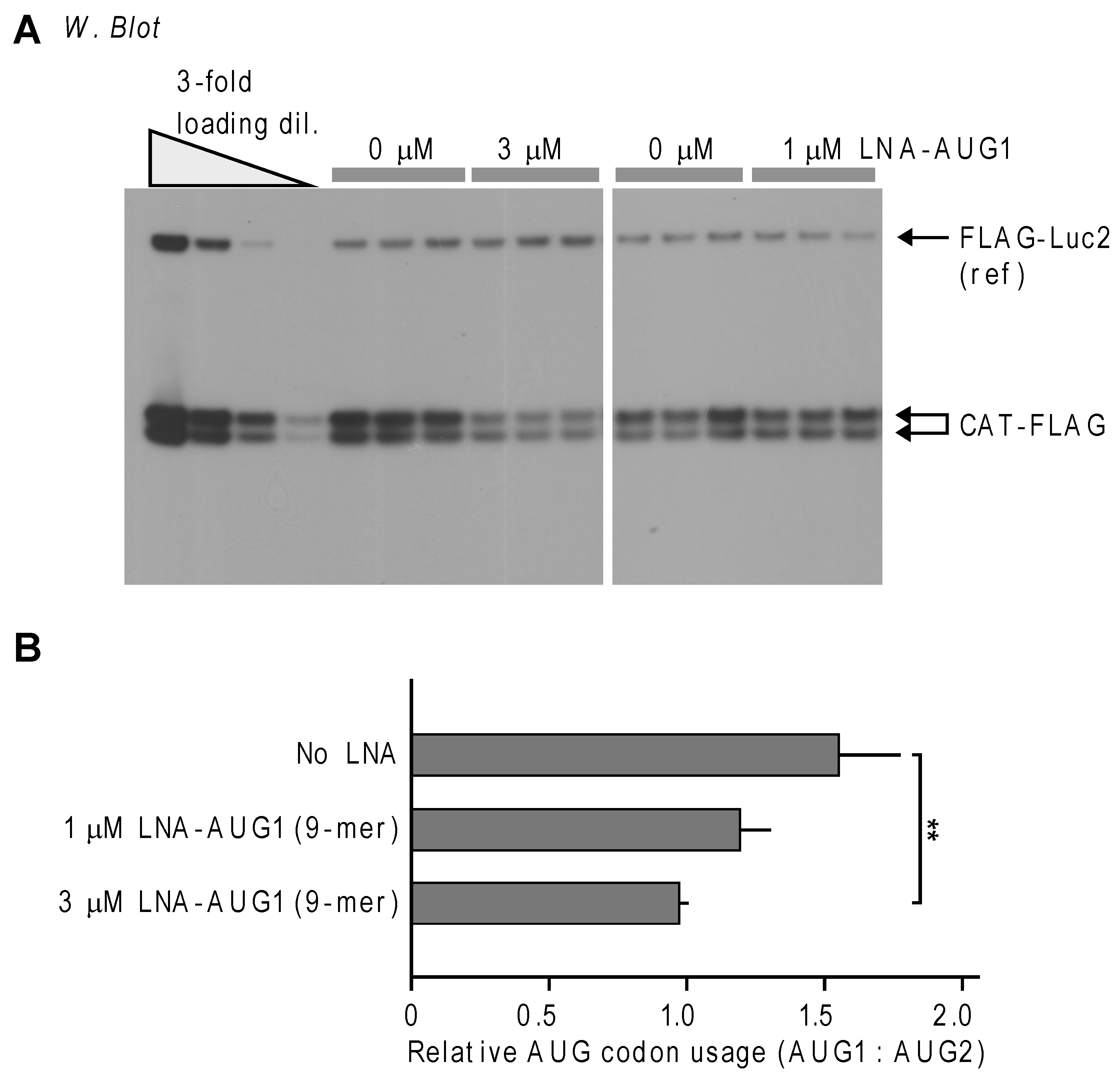

Supplement: Figure S2 — 9-nt LNA-AUG1 modulates translation of the target mRNA with minimal inhibitory effect on AUG2. (A) A 9-mer LNA oligonucleotide that targets AUG1 was cotransfected into COS-7 cells with plasmids expressing (CAA)4 CAT-FLAG and FLAG-Luc2 mRNAs. Two different concentrations of the LNA oligonucleotide were tested: 1 µM and 3 µM. (B) The relative protein expression (AUG1:AUG2) was quantified as in Figure 2 and plotted as a histogram, with error bars indicating standard deviations. Three independent experiments were performed to calculate the relative protein expression. Asterisks indicate statistically significant differences (** one-sided t-test: p<0.01). (TIF) [file pone.0015057.s002.tif]

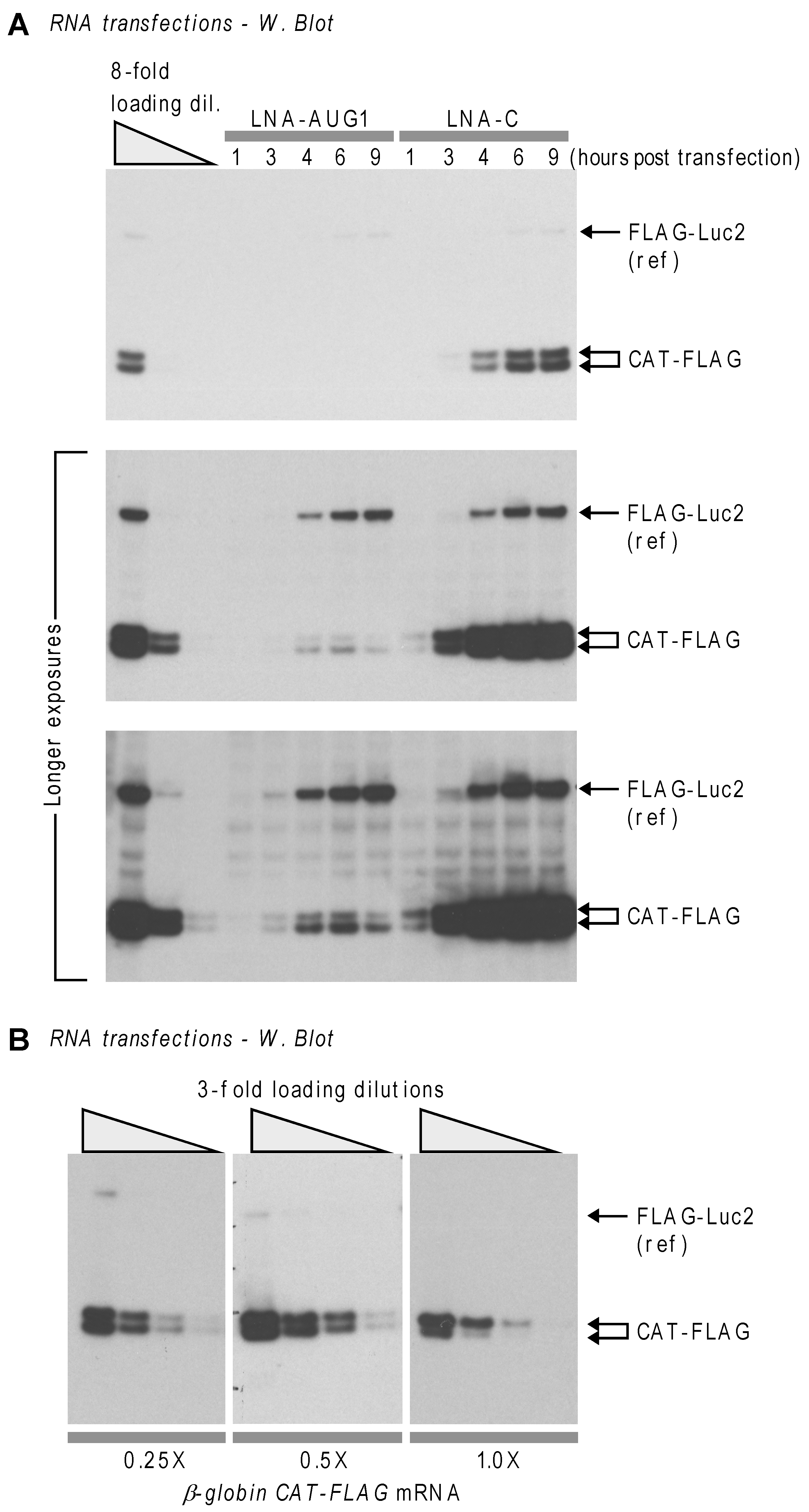

Supplement: Figure S3 — RNA transfections in COS-7 cells. (A) Time course of COS-7 cells transfected with in vitro transcribed 0.25X β-globin CAT-FLAG and FLAG-luc2 mRNAs. mRNAs were 5′-capped and poly(A)70-tailed in vitro transcripts (1 pmol each). Growth media was exchanged 1 hour post transfection and cells harvested at times indicated. An equal volume of cellular lysate was loaded in each lane of SDS-PAGE, along with 8-fold serial dilutions of the lysate. The expressed proteins were detected by anti-FLAG monoclonal antibody. Three different film exposures are shown. (B) Effects of 5′ leader length on AUG-codon usage in in vitro transcribed mRNAs. COS-7 cells were transfected with 5′-capped and poly(A)70-tailed in vitro transcripts (0.25X, 0.5X or 1.0X β-globin CAT-FLAG and FLAG-Luc2; 1 pmol each). Cells were harvested 5 hours post transfection. Three-fold dilutions of cell lysates were analyzed by Western blot using anti-FLAG monoclonal antibody. (TIF) [file pone.0015057.s003.tif]

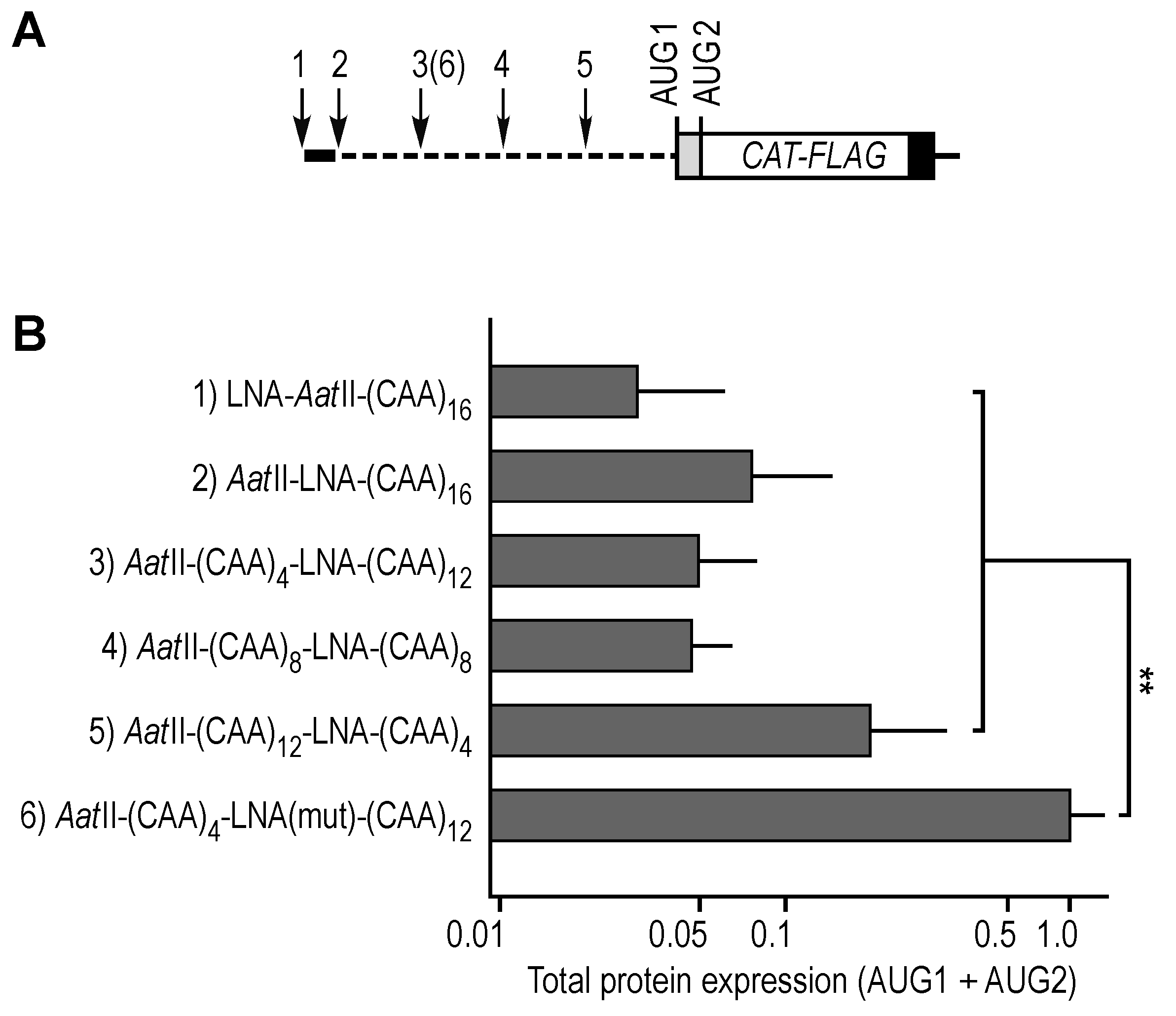

Supplement: Figure S4 — Effects of binding an LNA-oligonucleotide to different sites in the 5′ leader. (A) Schematic representation of constructs. The arrows (numbered 1-5) indicate the positions of individual LNA target sequences in the 5′ leaders of different constructs. Arrow 6 indicates the position of a mutated LNA target sequence. The thick black bar at the 5′ end of the mRNA represents an AatII sequence; the dashes in the 5′ leader represent CAA tri-nucleotide sequences. (B) COS-7 cells were transfected with 1 pmol each of 5′-capped and poly(A)70-tailed in vitro transcripts of FLAG-Luc2 and (CAA)16 CAT-FLAG mRNA variants that differ in the location of an LNA target site in the 5′ leader. 10 pmol of LNA-C, which is complementary to the LNA target sites in the 5′ leaders, was preincubated with the mRNA solutions before transfection. Total protein expression from AUG1 and AUG2 was quantified from Western blots by using the FLAG-Luc2 protein as a reference and plotted in a histogram, as a fraction of CAT-FLAG expression from an equivalent mRNA transfection, but without preincubation with LNA-C. Three independent experiments were performed for final quantification of the immunoblot with error bars indicating standard deviations. Asterisks indicate statistically significant differences with construct 6 (one-sided t-test: ** p<0.01). (TIF) [file pone.0015057.s004.tif]

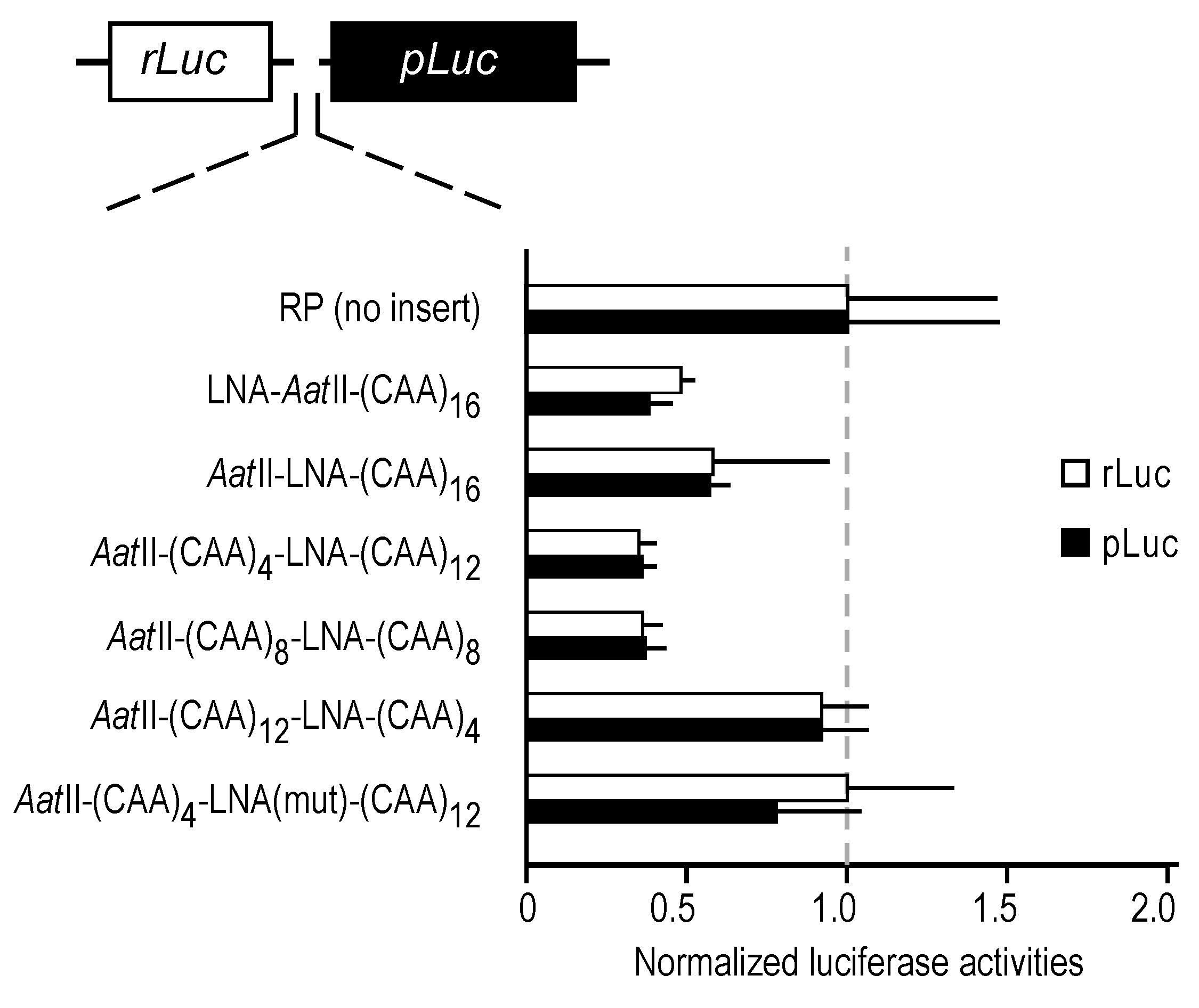

Supplement: Figure S5 — Dicistronic mRNA analysis of 5′ leaders with LNA binding sites at various locations. Sequences upstream of AUG2 in the (CAA)16 CAT-FLAG mRNA variants were tested in the intercistronic region of the Renilla/Photinus dual luciferase dicistronic mRNA for IRES activity as in Figure 1D. Vector sequences in the parent (RP) construct were used as a negative control. The results were plotted in a bar graph relative to the Renilla (rLuc) and Photinus luciferase (pLuc) activities from RP, which are individually defined as 1. Three independent experiments were performed for final quantification with error bars indicating standard deviations. (TIF) [file pone.0015057.s005.tif]
