# Supplementary material for: Determinants of Initiation Codon Selection during Translation in Mammalian Cells
Source: PLoS One. 2010 Nov 24;5(11):e15057. doi: 10.1371/journal.pone.0015057 (PMC2991327; doi:10.1371/journal.pone.0015057)
Supplement: Methods S1 — (DOC) [file pone.0015057.s006.doc]

**Supplementary Methods**

*Nucleotide sequences of 5' leaders* **−** (CAA)n (n=4, 10 or 16) 5' leaders contain an *Spe*I restriction site 5' of the CAA repeats. PCR products containing these sequences were cloned into pGL4-CAT-FLAG vector using *Bgl*II and *Hind*III restriction sites. The two ATG codons in the construct are underlined. The sequence ACTAGT is a recognition site for the *Spe*I restriction enzyme.

ACTAGTT-(CAA)n-CAGAAGATGGGTCGTGAGGCACTGGGCAGGTGTCCACTCCCAGG

TTCAATACAGCTCTTAAGCGGCCGCAAGCTTGCCGCCACCATGG

The amino acid sequence from AUG1 to AUG2 is as follows: MGREALGRCPLPGSIQLLSGRKLAATM.

*β -globin 5' leaders* **−** These constructs contain an *Aat*II restriction site 5' of the *β -globin* sequences. These sequences were cloned into pGL4-CAT-FLAG vector. ATG1 is underlined. The sequence GACGTC is a recognition site for the *Aat*II restriction enzyme.

0.25X β - *globin*

TC-GACGTC-AGAAACAGACATC-CAGAAGATGG

0.5X *β - globin*

TC-GACGTC-GACTCACAACCCCAGAAACAGACATC-CAGAAGATGG

1.0X *β - globin*

TC-GACGTC-ACATTTGCTTCTGACATAGTTGTGTTGACTCACAACCCCAGAAACA

GACATC-CAGAAGATGG

*(CAA)_16_ 5' leaders containing LNA-C binding sites* **−** These 5' leaders were cloned upstream of CAT-FLAG (no intron)-p(A)_70_; T7 RNA polymerase promoter directs transcription *in vitro*. The asterisk indicates the positions of LNA-C binding sites. The LNA mismatch sequence is used as a control, and is located at the third asterisk. The sequence GACGTC is a recognition site for the *Aat*II restriction enzyme. ATG1 is underlined.

GG-*-TC-GACGTC-*-CAACAACAACAA*CAACAACAACAA*CAACAACAACAA*CAACAACAACAA-CAGAAGATGG

LNA binding sequence = CAGTAGGAAGTCG

LNA mismatch sequence = CAGATCCTTCTCG

*Constructs for EJC experiments* **−** The 5' leader sequence is derived from pCAT3c. The asterisk indicates the location of the chimeric intron, which leads to EJC deposition 20-24 nucleotides upstream of the 5' end of the intron upon splicing. The two ATG-codons are underlined.

TCGGCCGCCTCGGCCTCTGAGCTATTCCAGAAGTAGTGAGGAGGCTTTTTTGGAGGCCTAGGCTTTTGCAAAAAGCTTGCGGCCGCTTAAGCTGCAGAAGATGGGTCGTGAGGCACTGGGCAG*GTGTCCACTCCCAGGTTCAATACAGCTCTTAAGCGGCCGCAAGCTTGCCGCCACCATGGAGAAAAAAATCACAG*GATATACCACCGTTGATATATCCCAATGGCATCGTAAAGAACATTTTCAG*G

Chimeric intron sequence

(GTAAGTATCAAGGTTACAAGACAGGTTTAAGGAGACCAATAGAAACTGGGCTTGTCGAGACAGAGAAGACTCTTGCGTTTCTGATAGGCACCTATTGGTCTTACTGACATCCACTTTGCCTTTCTCTCCACAG)

5' hairpin sequence

The complementary sequences that form the stem of a hairpin structure are underlined.

GACTCCTTGATAGATCTGGTACCGAGCTCCCCGGGCTGCAGCCCGGGGAGCTCGGTACCAGATCTATCAAGCTCTAGCCACCATCTCGAGCTCAAGCTTAAAACTAGTCAGCTGGATTC

# *Primer details* − Primer extension reactions used two primers: 5'-GAAAATGTTCTTTACGATGCC-3' and 5'-TATCCTGTGATTTTTTTCTCC-3' to anneal to sequences 67- and 23-nucleotides downstream of AUG2, respectively. Primers were used interchangeably to confirm the specific termination locations of primer extension reactions. Primers were 5'-end labeled with [γ-^32^P] ATP. The reverse transcription products were resolved in 6% denaturing gels and visualized using a Storm 860 PhosphorImager (GE Healthcare).

For semi-quantitative PCR, three primer pairs were used to detect *CAT-FLAG* mRNA variants:

5'-CACTAGTTCAACAACAACAACAACAG-3' and 5'-AAGCATTCTGCCGACATGGA-3', which generate a 719 bp product were used to detect the (*CAA*)_4_ *CAT-FLAG* mRNA.

5'-ATCGTAAAGAACATTTTCAGGCAT-3' and 5'-TAGAAACTGCCGGAAATCGTC-3', which generate a 302 bp product were used to detect all *CAT-FLAG* mRNA variants.

5'-TTCCAGAAGTAGTGAGGAGGCTT-3' and 5'-AAGCATTCTGCCGACATGGA-3', which generate a 769 bp product were used to detect mRNAs in the EJC experiment.

Primer pairs used to detect *FLAG-luc2* mRNA were: 5'-GTGGCCCCAGCTAACGACA-3' and

5'-CCGGGTGGCAAATGGGAAGT-3', which generate a 221 bp product; and

5'-GTGTTGGGTGCCCTGTTCATC-3' and 5'-GGGCATGACTGAATCGGACA-3', which generate a 394 bp product. In parallel, serial dilutions of RNA samples were analyzed by RT-PCR to allow quantification of the relative abundance of each mRNA construct.

*Probes for Northern Blots* **−** The sequences of the upstream probes, which hybridize to the *β-globin* 5' leader are 5'-GTGAGTCAACACAACTATGTCAGAAGCAAATGTGA-3' and

5'-ATGTCTGTTTCTGGGGTT-3'. The sequences of the downstream probes, which hybridize to the coding region of CAT-FLAG gene are 5'-TATCCTGTGATTTTTTTCTCC-3' and

5'-GAAAATGTTCTTTACGATGCC-3'. Northern blot hybridizations were visualized using a Storm 860 PhosphorImager (GE Healthcare). For detection of the transiently expressed *FLAG-luc2* control mRNAs on the same membranes, ^32^P end-labeled probes (5'-GGGCATGACTGAATCGGACA-3' and

5'-CCGGGTGGCAAATGGGAAGT-3') were subsequently hybridized to the coding region using the same conditions used for the detection of *CAT-FLAG* mRNAs.
